# The Effects of Prolonged Basic Amino Acid Exposures on Mitochondrial Enzyme Gene Expressions, Metabolic Profiling and Insulin Secretions and Syntheses in Rat INS-1 β-Cells

**DOI:** 10.3390/nu15184026

**Published:** 2023-09-17

**Authors:** Lianbin Xu, Fengqi Cheng, Dengpan Bu, Xiuli Li

**Affiliations:** 1 College of Animal Science and Technology, Qingdao Agricultural University, Qingdao 266109, China; xulianbin@qau.edu.cn (L.X.); 20222103003@stu.qau.edu.cn (F.C.); budengpan@qau.edu.cn (D.B.); 2 College of Veterinary Medicine, Qingdao Agricultural University, Qingdao 266109, China

**Keywords:** basic amino acid, insulin release, INS-1 β-cells, metabolic profiling, mitochondrial enzyme, metabolism-secretion coupling

## Abstract

In order to investigate the chronic effects of basic amino acids (BAA) on β-cell metabolism and insulin secretion, INS-1 β-cells were randomly assigned to cultures in standard medium (Con), standard medium plus 10 mM L-Arginine (Arg), standard medium plus 10 mM L-Histidine (His) or standard medium plus 10 mM L-Lysine (Lys) for 24 h. Results showed that insulin secretion was decreased by the Arg treatment but was increased by the His treatment relative to the Con group (*p* < 0.05). Higher BAA concentrations reduced the high glucose-stimulated insulin secretions (*p* < 0.001), but only Lys treatment increased the intracellular insulin content than that in the Con group (*p* < 0.05). Compared with Arg and Lys, the His treatment increased the mitochondrial key enzyme gene expressions including *Cs*, *mt-Atp6*, *mt-Nd4l* and *Ogdh*, and caused a greater change in the metabolites profiling (*p* < 0.05). The most significant pathways affected by Arg, His and Lys were arginine and proline metabolism, aminoacyl-tRNA biosynthesis and pyrimidine metabolism, respectively. Regression analysis screened 7 genes and 9 metabolites associated with insulin releases during BAA stimulations (*p* < 0.05). Together, different BAAs exerted dissimilar effects on β-cell metabolism and insulin outputs.

## 1. Introduction

Amino acids (AA) affect insulin secretion from pancreatic β-cells under appropriate conditions [1], and circulating concentrations of several AAs were found to be increased both in diabetic participants [2] and an insulin resistance state [3], suggesting the critical role of abnormal AA metabolism in the pathogenic mechanism of diabetes.

A previous study showed that the plasma insulin response in humans was dependent on the rate of appearance of AA after the ingestion of different protein solutions [4]. Further evidence indicated that the effects of different AAs on insulin releases both in primary islets and pancreatic β-cells are dependent on the AA category [5]. Glutamine, alanine and arginine were reported to increase glucose-stimulated insulin secretions (GSIS), indicating that β-cell AA and glucose metabolism share common pathways [6]. To sum up, AAs affect the triggering and amplification processes of insulin release partly through 3 pathways including (i) serving as a substrate for the TCA cycle and/or redox shuttles with the subsequent generation of ATP, (ii) a direct depolarization of the plasma membrane via transport of positively charged AAs into the cell and (iii) the depolarization of the plasma membrane via co-transport of Na^+^ ions along with the AA into the cell [5,7].

Previous work established that a single AA at physiological concentrations may not affect GSIS, but supplied in specific combinations at physiological concentrations or individually at higher concentrations can promote GSIS [6]. Among all AAs, the insulinotropic actions of basic AAs (BAA) are currently ascribed to the accumulations of these positively charged molecules in β-cells, resulting in the depolarization of the plasma membrane and subsequent gating of voltage-sensitive Ca^2+^ channels [8]. However, Sener et al. found that the secretory response of β-cells to L-Lysine was different from that stimulated by other cationic AAs, which may be partly explained by the capacity of L-Lysine to act as a fuel in islet cells [9]. Therefore, the metabolizability of various BAAs in β-cells and their associations with insulin responses need to be further investigated.

The metabolism of various fuel stimuli in β-cells is necessary for nutrient-induced insulin secretion [10]. Specifically, mitochondria establish numerous links to the plasma membrane channels, cell redox, NADPH and Ca^2+^ homeostasis, which all have important effects on insulin secretion [11]. Nutrients acting as a substrate for a TCA cycle depend on a series of mitochondrial key enzymes, such as acetyl-CoA carboxylase, citrate synthase, oxoglutarate dehydrogenase and so on [5,10,12]. In addition, several transcription factors are also important for the maintenance of mitochondrial functional homeostasis. Pancreas/duodenum homeobox protein 1 (PDX1) serves as a marker of the developing pancreas and participates in controlling the β-cell identity [13], while transcription factor A (TFAM) offers a molecular foundation for the link between external stimuli and mitochondrial biogenesis [14]. Therefore, investigating the effects of BAAs on mitochondria key enzyme and transcription factor expressions will contribute to understanding the BAA-induced metabolism-secretion coupling.

The objectives of this study were to investigate the metabolic profiling and mitochondrial metabolism of INS-1 β-cells exposed to 3 different BAAs and evaluate their associations with insulin outputs.

## 2. Materials and Methods

### 2.1. Cell Culture and Treatments

Rat INS-1 β-cells that were provided by the Scientific Center, Shandong Provincial Hospital affiliated to Shandong University, were cultured in an RPMI 1640 medium (Gibco, NY, USA) including 11.11 mM glucose, 50 μmol/L β-mercaptoethanol (Sigma-Aldrich, St. Louis, MO, USA), 10% (*v*/*v*) FBS (Gibco, NY, USA), 100 IU/mL penicillin (Solarbio, Beijing, China) and 100 mg/L streptomycin (Solarbio, Beijing, China) at 37 °C with 5% CO_2_.

Previous works reported that 10 mmol/L AA in a medium generated a strong stimulus to insulin release [15,16]. For investigating the chronic effects of various BAA administrations, β-cells were cultured in multi-well plates to allow overnight adhesion. Then, the medium was discarded, and the cells were randomly allocated to one of the following treatments where the concentration of L-Arginine (Arg), L-Histidine (His) or L-Lysine (Lys) was increased to 10 mmol/L and the remaining components were kept the same as the standard medium (Con). For the preparation of treatment media, L-Arginine, L-Histidine or L-Lysine (Sigma-Aldrich, St. Louis, MO, USA) was dissolved in an RPMI 1640 medium (Gibco, NY, USA), adjusted to a pH of 7.2, and passed through a 0.22-μm sterile filter membrane (Millipore Sigma, Burlington, MA, USA). Cell samples were collected after a 24 h treatment and stored at −80 °C for further analysis.

### 2.2. Cell Proliferation Assay

After the individual BAA treatment, β-cells were grown in new medium including 10 μL/well CCK-8 reagent (Beyotime Biotechnology Co., Ltd., Shanghai, China) and then maintained at 37 °C for 2 h. The ELX808 microplate plate reader (Biotek, Winooski, VT, USA) was used to determine the absorbance of dissolved solutions at 450 nm that were adjusted to the results from a well that was only added with a growth medium.

### 2.3. Insulin Release and GSIS Assay

Insulin concentrations in the test medium and cell lysate were measured by ELISA using the commercial kits (Shanghai MLBIO Biotechnology Co., Ltd., Shanghai, China). For GSIS, the INS-1 β-cells were first treated with individual BAA for 24 h. Then, the cells were preincubated in a KRBH buffer (Solarbio, Beijing, China) containing 0.5% BSA and 1.1 mM glucose for 0.5 h. After that, the cells were treated with fresh KRBH buffer including 1.1 or 16.7 mM glucose for 1 h. The insulin concentration in supernatant of each well was adjusted to the number of β-cells.

### 2.4. Glucose Uptake and ATP/ADP Ratio Assay

Glucose uptakes in INS-1 β-cells during BAA administrations were measured as described by Kiely et al. [17]. Briefly, the glucose concentrations in RPMI-1640 at the start and end of the administration were determined by the Glucose Assay Kit (Maccura Biotechnology Co., Ltd., Sichuan, China). The glucose uptake over the treatment period was evaluated by subtracting the concentration at 24 h from that at 0 h, which was normalized to the total protein contents in the INS-1 β-cells. For the ATP/ADP ratio assay, INS-1 β-cells were seeded in black 96 well plates and treated with BAAs for 24 h. Following the administration, the ATP and ADP concentrations were determined with the firefly luciferase in the ATP/ADP Ratio Assay Kit (Sigma-Aldrich, St. Louis, MO, USA), following the manufacturer’s instructions.

### 2.5. Real-Time Quantitative PCR

TRIzol reagent (Invitrogen, Carlsbad, CA, USA) was used to extract the total RNA of treated cells. The quantity and quality of RNA were measured using a biophotometer (Eppendorf, Hamburg, Germany). Reverse transcription was performed with a reaction buffer including 500 ng of total RNA, 0.5 μL PrimeScript RT Enzyme Mix I, 2 μL 5× PrimeScript Buffer, 25 pmol Oligo dT and 50 pmol Random 6 mers (Takara Bio-Company, Shiga, Japan). Ten nanograms of cDNA were amplified in 20 μL of PCR reaction buffer containing 0.2 μmol/L of each specific primer and an SYBR green master mix (Takara Bio-Company, Shiga, Japan). Real-time quantitative PCR was performed by using the 7500 Real-Time PCR System (Applied Biosystems, Waltham, MA, USA) with the SYBR Premix Ex Taq Kit (TaKaRa, Shiga, Japan), following the recommended instructions. *Actb* and *Gapdh* were used as the internal standards in the quantification, and the relative amount of mRNA was calculated according to the 2^−ΔΔCt^ formula. The primer sequences used in the qPCR were presented in Table 1.

### 2.6. Metabolomics Analysis

For the metabolic profiling, INS-1 cell samples (*n* = 6) were separated on an Agilent 1290 Infinity LC Ultra-High-Performance Liquid Chromatography System (Agilent Technologies, CA, USA) using a HILIC column. The column temperature was 25 °C, and the flow rate was 0.3 mL/min. The mobile phase of composition A included water, 25 mmol/L ammonium acetate and 25 mmol/L ammonia, and B was acetonitrile. Pooled quality control (QC) samples generated by taking an equal aliquot of all samples were run 3 times before the beginning of the sample queue for column conditioning and every 6 injections thereafter to assess inconsistencies. Electrospray ionization (ESI) in positive and negative ion modes were used for detection. The sample was separated by UHPLC and subjected to mass spectrometry using a TripleTOF 5600 mass spectrometer (AB SCIEX, Framingham, MA, USA). Non-targeted LC-MS/MS vendor raw data files were converted and processed by using the ProteoWizard MSconvert (Version 3.0) and XCMS online (Version 3.5.1), respectively. Further analysis of processed data was carried out by the MetaboAnalyst (Version 5.0) following the normalization and integration by using support vector regression. Variable importance in the projection (VIP) was calculated to evaluate the contribution of each variable to the classification in the orthogonal partial least-squares discriminant analysis (OPLS-DA) model. Significance was determined using an unpaired Student’s *t* test. A VIP value > 1 and *p* < 0.05 was considered statistically significant.

### 2.7. Metabolite–Metabolite Interaction Network Analysis

The metabolite–metabolite network of differentially expressed metabolites (DEM) was performed using Cytoscape software (Version 3.8.0) following the Spearman correlation analysis. Connections with |correlation coefficient| > 0.7 and *p* < 0.05 were considered significant. Hub metabolites in the network were distinguished according to the degree of connectivity.

### 2.8. Western Blotting

Ten micrograms of protein were separated by sodium dodecyl sulfate polyacrylamide gel electrophoresis, and then transferred to polyvinylidene fluoride membranes (Millipore, Billerica, MA, USA). The membranes were incubated in a blocking buffer (Beyotime Biotechnology Co. Ltd., Shanghai, China) for 2 h at room temperature and were incubated overnight at 4 °C with primary antibodies against Acetyl-CoA Carboxylase (#3676), Citrate Synthase (#14309) and Oxoglutarate Dehydrogenase (#26865) antibodies (Cell Signaling Technology, Danvers, MA, USA). The blots were then incubated with a secondary antibody (Beyotime Biotechnology Co., Ltd., Shanghai, China) for 2 h at room temperature. Relative band densities were determined using a Fusion Fx imaging system (Vilber Lourmat, Marne-la-Vallee, France). β-Actin and α-Tubulin expressions were used as internal controls.

### 2.9. Statistical Analysis

Results are shown as means ± SEMs. Statistical analyses were performed with the ANOVA of SAS software (version 9.2, SAS Institute Inc., Cary, NC, USA). Tukey’s HSD test was used to make a post hoc treatment comparison. Log-transformed data was applied for analysis when the variances of variables were unequal among all groups. The association between individual gene or DEM expression with insulin response was assessed by the multiple linear regression with backward stepwise elimination, and the multicollinearity among various parameters was evaluated by the variance inflation factor (VIF). Variables were removed one by one in turns until all variables left in the model tended to be significant (*p* < 0.1) and the VIF was < 10.

## 3. Results

### 3.1. Cell Proliferation, Insulin Release and GSIS

**Figure 1:**
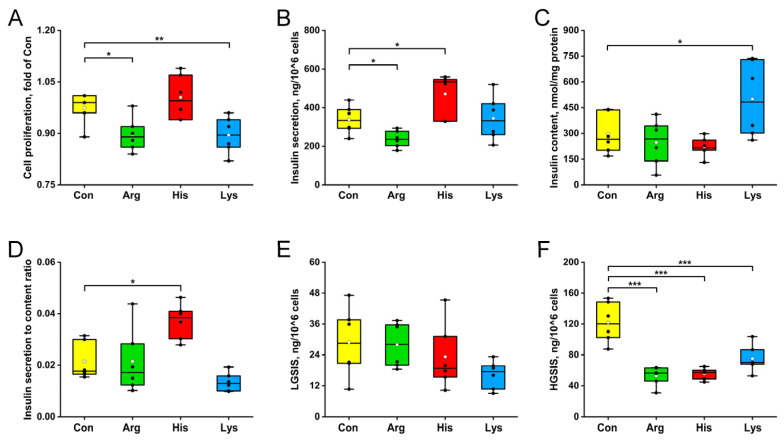
The effects of 10 mM different basic amino acids on cell proliferations (**A**), insulin secretions (**B**), insulin contents (**C**), insulin secretion to content ratios (**D**) and glucose-stimulated insulin secretions (**E**,**F**) in INS-1 β-cells after 24 h treatment. The black circle means the raw data, and the white circle represents the mean of data. *n* = 6. * *p* < 0.05, ** *p* < 0.01 and *** *p* < 0.001 relative to the Con group. HGSIS, high glucose-stimulated insulin secretion; LGSIS, low glucose-stimulated insulin secretion.

Elevated Arg and Lys concentrations significantly decreased the proliferations of INS-1 β-cells relative to the Con group (*p* < 0.05, Figure 1A). Insulin secretion was reduced by the Arg treatment but was increased by the His treatment than that with the Con treatment (*p* < 0.05, Figure 1B). Chronic Lys administration elevated the intracellular insulin content than that of the Con group (*p* < 0.05, Figure 1C). INS-1 β-cells cultured with 10 mM His had higher insulin secretion to content ratios (ISCR) than those cultured with the standard medium (*p* < 0.05, Figure 1D). For the GSIS, prolonged Arg, His and Lys exposures depressed the insulin responses of β-cells to 16.7 mM glucose stimulus (*p* < 0.001, Figure 1F), while the 1.1 mM glucose-stimulated insulin secretion was not influenced by the BAA administrations (*p* > 0.05, Figure 1E).

### 3.2. AA and Glucose Transports

**Figure 2:**
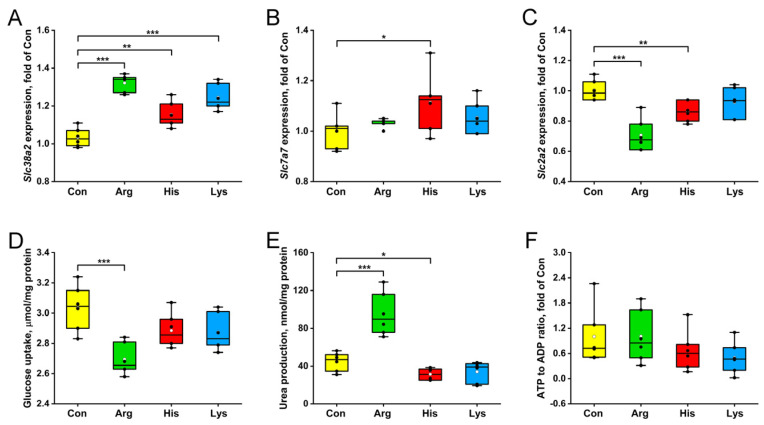
The effects of 10 mM different basic amino acids on the gene expressions of amino acid and glucose transporters (**A**–**C**), glucose uptakes (**D**), urea concentrations (**E**) and ATP to ADP ratios (**F**) in INS-1 β-cells after 24 h treatment. The black circle means the raw data, and the white circle represents the mean of data. *n* = 6. * *p* < 0.05, ** *p* < 0.01 and *** *p* < 0.001 compared with the Con group. ADP, Adenosine 5′-diphosphate; ATP, Adenosine 5′-triphosphate; *Slc2a2*, solute carrier family 2 member 2; *Slc7a7*, solute carrier family 7 member 7; *Slc38a2*, solute carrier family 38 member 2.

INS-1 β-cells with Arg, His and Lys treatments had higher expressions of the *Slc38a2* gene involved in neutral AA transport relative to the Con group (*p* < 0.01, Figure 2A), while only His administration increased the mRNA abundance of the *Slc7a7* gene responsible for BAA transmembrane transporter activity (*p* < 0.05, Figure 2B). Higher concentrations of Arg and His reduced the gene expression levels of the *Slc2a2* encoding glucose transporter (*p* < 0.01, Figure 2C), and Arg treatment inhibited the glucose uptakes of INS-1 β-cells than those of the Con group (*p* < 0.001, Figure 2D). Intracellular urea concentration was increased by the Arg treatment but was decreased by the His treatment relative to the Con group (*p* < 0.05, Figure 2E). No significant difference in the ATP to ADP ratio was identified among the 4 groups (*p* > 0.05, Figure 2F).

### 3.3. Mitochondrial Key Enzyme and Transcription Factor Expressions

**Figure 3:**
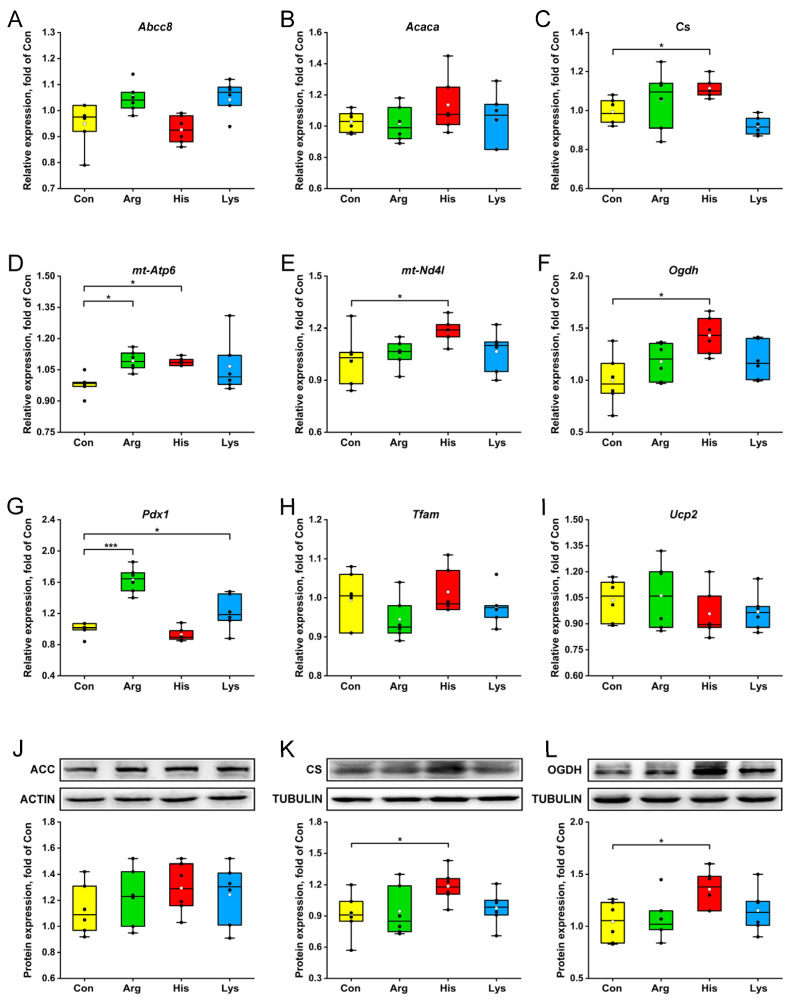
The effects of 10 mM different basic amino acids on the expressions of genes (**A**–**I**) and enzymes (**J**–**L**) involved in mitochondrial metabolism in β-cells after 24 h treatment. The black circle means the raw data, and the white circle represents the mean of data. *n* = 6. * *p* < 0.05 and *** *p* < 0.001 relative to the Con group. *Abcc8*, ATP binding cassette subfamily C member 8; *Acaca*, acetyl-CoA carboxylase alpha; ACC, acetyl-CoA carboxylase; *Cs*, citrate synthase; *mt-Atp6*, ATP synthase 6, mitochondrial; *mt-Nd4l*, NADH dehdrogenase 4L, mitochondrial; *Ogdh*, oxoglutarate dehydrogenase; *Pdx1*, pancreatic and duodenal homeobox 1; *Tfam*, transcription factor A, mitochondrial; *Ucp2*, uncoupling protein 2.

Elevating Arg concentration on the basis of the standard medium increased the abundances of *mt-Atp6* and *Pdx1* mRNA, whereas His treatment up-regulated the gene expressions of *Cs*, *mt-Atp6*, *mt-Nd4l* and *Ogdh* (*p* < 0.05, Figure 3C–G). Relative to those in the Con group, *Pdx1* transcriptions in INS-1 β-cells were promoted by the Lys administration (*p* < 0.05, Figure 3G). However, the expression levels of *Abcc8*, *Acaca*, *Tfam* and *Ucp2* genes were not affected by the BAA treatments (*p* > 0.05, Figure 3A,B,H,I). The intracellular acetyl-CoA carboxylase, citrate synthase and oxoglutarate dehydrogenase concentrations were measured by western blotting to validate the accuracy of qPCR, and there was an agreement in their expressions between gene and protein levels (Figure 3J–L).

### 3.4. Metabolomics Analysis

**Figure 4:**
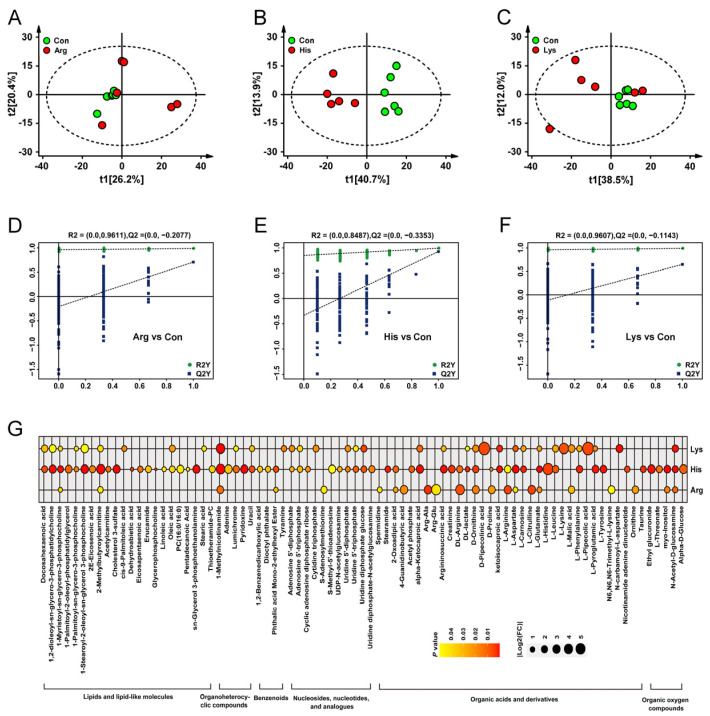
The metabolomics analyses of INS-1 β-cells treated with different basic amino acids, *n* = 6. PCA (**A**–**C**) and OPLS-DA (**D**–**F**) plots of metabolites in INS-1 β-cells treated with different basic amino acids. The relative expressions of DEMs between individual basic amino acid administrations and the Con group in INS-1 β-cells (**G**). DEMs, differentially expressed metabolites; FC, fold change; OPLS-DA, orthogonal partial least-squares discriminant analysis; PCA, principal component analysis.

Combined with VIP value and statistical analysis, 22, 64 and 36 DEMs triggered by Arg, His and Lys treatments, respectively, were identified relative to the Con group (Figure 4G). Principal components analysis (PCA) revealed a marked separation between His vs. Con comparison, while Arg and Lys stimulations had overlaps with the Con group (Figure 4A–C). The OPLS-DA plots showed that the model was reliable and there was not an overfitting (Figure 4D–F). The relative expressions of DEMs among 3 groups are shown in Figure 4G and Supplementary Materials Table S1. A horizontal comparison indicated that 7 metabolites, including 1-Myristoyl-sn-glycero-3-phosphocholine, D-Ornithine, L-Aspartate, L-Glutamate, N-Acetyl-D-glucosamine, 2-Methylbutyroylcarnitine and 1-Methylnicotinamide, were affected by all 3 BAA administrations (*p* < 0.05).

**Figure 5:**
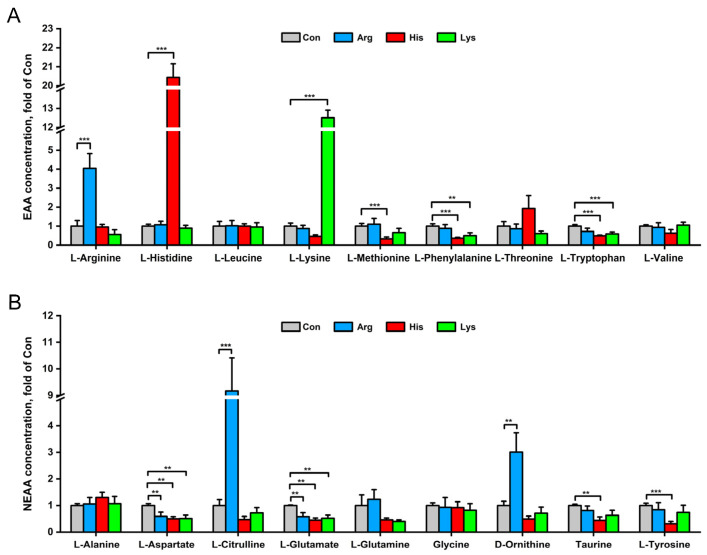
The effects of 10 mM different basic amino acid treatments on intracellular concentrations of EAA (**A**) and NEAA (**B**) in INS-1 β-cells after 24 h treatment. Values are means ±SEMs, *n* = 6. ** *p* < 0.01 and *** *p* < 0.001 relative to the Con group. EAA, essential amino acid; NEAA, nonessential amino acid.

Metabolomic data showed that Arg treatment increased intracellular L-Arginine, L-Citrulline and D-Ornithine concentrations, but decreased L-Aspartate and L-Glutamate concentrations than those in the Con group (*p* < 0.01, Figure 5). Despite the elevated L-Histidine concentration, His exposure significantly reduced the intracellular concentrations of 3 essential AAs and 4 nonessential AAs, including L-Methionine, L-Phenylalanine, L-Tryptophan, L-Aspartate, L-Glutamate, Taurine and L-Tyrosine (*p* < 0.01, Figure 5). Similar to the His treatment, Lys administration also decreased the L-Phenylalanine, L-Tryptophan, L-Aspartate and L-Glutamate concentrations (*p* < 0.01, Figure 5).

### 3.5. Hub Metabolite and KEGG Pathway

**Figure 6:**
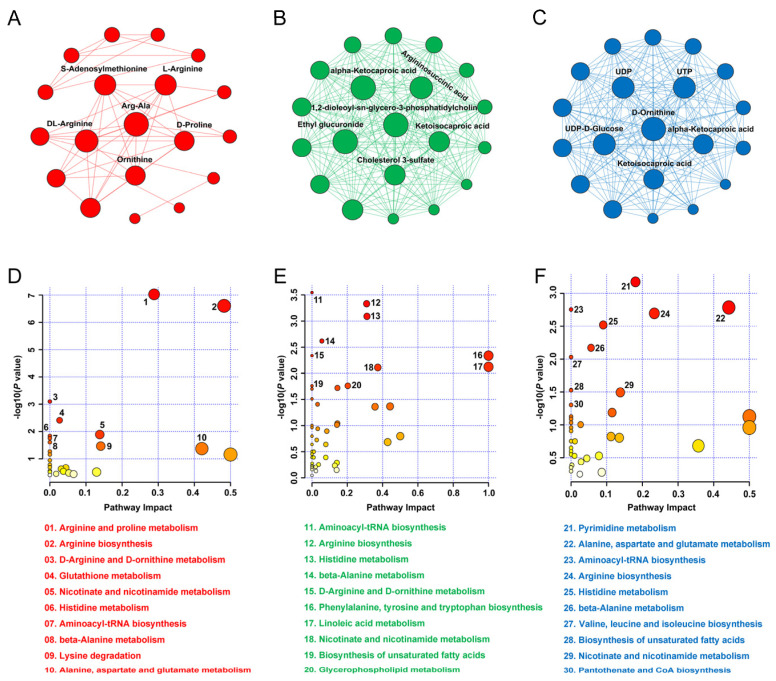
The interaction network of DEMs induced by different basic amino acids (**A**–**C**). The size of each node is positively related to the number of the degree, and the top 6 metabolites having the highest degrees were labeled with names. The pathway enrichments of DEMs between individual basic amino acid treatments and the Con group in 4 INS-1 β-cells (**D**–**F**). DEMs, differentially expressed metabolites.

A total of 19, 63 and 36 nodes as well as 48, 1026 and 237 edges were included in the metabolite–metabolite interaction network of INS-1 β-cells based on the DEMs induced by the Arg, His and Lys administrations, respectively (Figure 6A–C). By accessing the degree centrality of network, the hub metabolite in the network induced by Arg, His or Lys treatments was Arg-Ala, 1,2-dioleoyl-sn-glycero-3-phosphatidylcholine and D-Ornithine, respectively (Figure 6A–C). Enrichment analysis demonstrated that the most significant pathway affected by Arg, His and Lys treatments was arginine and proline metabolism, aminoacyl-tRNA biosynthesis and pyrimidine metabolism, respectively (Figure 6D–F). Comparative analysis showed that arginine synthesis, nicotinate and nicotinamide metabolism, histidine metabolism, aminoacyl-tRNA biosynthesis and beta-alanine metabolism were the 5 pathways affected by all 3 BAA administrations (Figure 6D–F).

### 3.6. Key Genes and Metabolites Related to Insulin Responses

When combining the groups, multiple regression analyses revealed that the insulin secretions were mainly correlated with *Slc7a7*, *Pdx1* and *Tfam* gene expressions (R^2^ = 0.67), while the intracellular insulin contents were mainly correlated with *Cs* and *Nd4l* gene expressions (R^2^ = 0.32, Table 2). On the other hand, the mRNA abundances of *Slc38a2* and *Ogdh* explained 53% of the variation in high glucose-stimulated insulin secretion (HGSIS; *p* = 0.0009 and *p* = 0.011, respectively), whereas *Cs* and *Slc7a7* were the genes most closely associated with ISCR (R^2^ = 0.24) and low glucose-stimulated insulin secretion (LGSIS; R^2^ = 0.26), respectively (Table 2).

With respect to the DEMs, Argininosuccinic acid, 1,2-dioleoyl-sn-glycero-3-phosphatidylcholine and oleic acid was the only one metabolite left in the multiple regression model that was associated with insulin secretion (R^2^ = 0.29), ISCR (R^2^ = 0.28) and LGSIS (R^2^ = 0.17), respectively (Table 3). In addition, intracellular concentrations of L-Citrulline, 1-Methylnicotinamide, 1,2-Benzenedicarboxylic acid, Alpha-D-Glucose, Linoleic acid and Dehydroabietic acid collectively explained 89% of the variability in HGSIS (all *p* < 0.05).

## 4. Discussion

The knowledge of the mechanisms linking different BAA metabolisms with insulin processing in β-cells is relatively lacking, and the identification of key genes and metabolites as well as signaling pathways would prove invaluable for antidiabetic targets. This study systematically compared the mitochondrial metabolism and metabolic profiling of INS-1 cells between different BAA treatments, and evaluated their associations with insulin secretions, intracellular insulin contents and GSIS, which will contribute to understanding the BAA-induced metabolism-secretion coupling and exploring attractive biomarkers or effective therapeutic targets for diabetes mellitus.

### 4.1. Effects of High BAA Treatment on β-Cell Metabolism

The PCA score plots between Arg vs. Con, His vs. Con and Lys vs. Con comparisons revealed the diverse metabolic patterns of β-cells exposed to different BAA administrations. Among the 3 BAAs, 10 mM L-Histidine may have the most significant effects on INS-1 β-cell metabolism, which was supported by the greater number of DEMs and larger changed expressions of mitochondrial key enzyme genes. Similar to a previous report that studied how BAAs inhibited the degradation of phospholipids and lipid oxidation [18], our data showed that Arg, His and Lys treatments reduced the intracellular concentration of 1-Myristoyl-sn-glycero-3-phosphocholine that is a lysophosphatidylcholine 14:0 in which the acyl group specified is myristoyl; this highlighted the association between BAA availability and lipid metabolism. L-Glutamate and L-Aspartate, critical intermediates coupling mitochondrial metabolism to insulin secretion [19,20], were also simultaneously affected by 3 BAA treatments, suggesting the links between different BAA metabolisms. Metabolism is usually the primary way by which an excess of AAs is controlled, but transport is critical for translating intake into higher cytosolic AA concentrations [21]. Therefore, the higher supply and intracellular concentrations of BAAs may inhibit other AA imports, resulting from the limited capacity of the transporter, and then decrease other AA concentrations. However, contrary to His, excessive Arg and Lys supplies had no remarkable effects on the expressions of genes encoding the mitochondrial key enzymes. It is likely, but not demonstrated, that the insulin responses to excessive Arg and Lys administrations were mainly due to the disturbed flux of nutrients rather than mitochondrial activity, but the underlying mechanism needs to be further explored.

### 4.2. Insulin Response to Excessive Arginine

Our present data showed that high concentrations of different BAAs exerted various effects on insulin secretions in INS-1 β-cells, emphasizing the prior finding that divergent effects on insulin releases took place depending on the AA type [6]. Consistent with previous studies that stated that chronic L-Arginine stimulation impeded insulin secretion [22,23], we found that 10 mM Arg treatment for 24 h decreased insulin output and HGSIS. Pancreatic β-cells respond to plasma calorigenic nutrient levels and modulate insulin secretion in light of the needs of the organism, and glucose is the primary insulin stimulus [10]. The reduced gene expressions of the *Slc2a2* encoding glucose transporter and decreased glucose uptake in our current study partly explained the lower insulin response of INS-1 β-cells to Arg treatment. One possible mechanism underlying this observation was reported to be that excessive nitric oxide generated from L-Arginine-decreased glycolysis in hepatocytes by reducing the activities of glucokinase and glyceraldehyde-3-phosphate dehydrogenase to decline glucose utilization [24].

On the other hand, our current data showed that chronic Arg administration decreased cell proliferation, which was supported by a previous study that stated that a high concentration of L-Arginine increased the number of apoptotic cells and gene expressions related to endoplasmic reticulum stress [22], suggesting that excessive L-Arginine exposure damaged β-cell activity and then resulted in decreased insulin secretion.

### 4.3. Insulin Response to Excessive Histidine

Regarding the effects of L-Histidine on insulin secretion, Parkash et al. reported that L-Histidine inhibited calcium channel activity and insulin output in β-cells [25], while Sjöholm et al. found that L-Histidine dose-dependently elevated insulin output without regulating the insulin content of islets [26], and we observed that a high concentration of L-Histidine elevated the insulin output and insulin release rate calculated as ISCR. This finding was confirmed by a previous observation that a significant synergistic effect was exerted by L-Histidine and glucose combinations when infused intravenously to healthy men [27]. Barisón et al. reported that L-Histidine provided electrons to the electron transport chain as an energy source in Trypanosoma cruzi, efficiently keeping mitochondrial membrane potential [28]. Therefore, one explanation for the insulinotropic action of His is the strengthened β-cell mitochondrial metabolism, as reflected by the up-regulated gene and protein expressions of key enzymes involved in the TCA cycle including *Cs* and *Ogdh*. Citrate, a product of citrate synthase, is a critical regulatory metabolic coupling factor that is involved in amplifying the pathways of fuel-stimulated insulin secretion [10]. Our regression analysis confirmed that *Cs* gene expression in a β-cell explained 24% of the variability in ISCR. A high expression level of *Ogdh* that encodes one subunit of the 2-oxoglutarate dehydrogenase complex may strengthen the catabolism of α-Ketoglutaric acid to a NADH that participates in affecting insulin output in response to glucose stimulation [29].

His may also mediate insulin output through affecting other AA transports. A previous study conducted in 5181 Finnish men confirmed that 9 AAs were significantly correlated with decreased insulin secretion and increased glucose concentrations [30]. In our current study, His treatment increased the *Slc7a7* mRNA abundance relative to the Con group. LAT1, encoded by *Slc7a7*, was observed to have higher expressions in pancreatic islets and to regulate neutral AA transport with a high affinity [31]. Kobayashi et al. further demonstrated that a reduced LAT1-mediated AA uptake contributed to the inhibited insulin release in β-cells [32].

Metabolomics analysis demonstrated that His administration had the most significant effect on metabolic profiling among the 3 BAAs. Further interaction network analysis revealed that 1,2-dioleoyl-sn-glycero-3-phosphatidylcholine was the hub metabolite of the DEMs induced by His treatment. This result suggested that a high concentration of L-Histidine may regulate insulin output through regulating the lipid metabolism of INS-1 β-cells, which was supported by the current data that His treatment increased the concentrations of oleic acid and linoleic acid that were reported to elevate insulin output in a glucose-dependent manner [33].

### 4.4. Insulin Response to Excessive Lysine

Among the 3 BAAs, Lys treatment increased intracellular insulin content without affecting insulin secretion, which was parallel to the report by Xu et al. that plasma L-Lysine concentration was the unique independent variable left in the multiple regression analysis model that had a strong positive correlation with the pancreatic insulin content in lactating rats [34]. PDX1 serves as a marker of the developing pancreas and binds to regulatory elements to increase insulin gene transcription [13]. The higher intracellular insulin content in *β*-cells treated with excessive Lys was partly ascribed to the up-regulated gene expression of *Pdx1*, but more details regarding the regulation of Lys on insulin synthesis need to be further investigated.

### 4.5. Key Genes and Metabolites Associated with Insulin Response

Multiple regression analyses were used to screen key genes and metabolites related to insulin responses during BAA treatments. When combining all groups, our data demonstrated that the gene expression of *Tfam* was correlated with insulin secretion and LGSIS. *Tfam*, which controls stability and transcriptional activity, was reported to participate in insulin secretion by coupling with the electron transport chain regulation [35]. In addition, *Cs* was one of the genes most closely related to the insulin content. Xu et al. observed that altered citrate synthase expression was involved in the ATF6 mediated endoplasmic reticulum function [36] that is critical for insulin synthesis and the folding of newly synthesized proinsulin [37].

Contrary to a previous finding that L-Argininosuccinate at physiological concentrations increased cytosolic Ca^2+^ concentration and resultant potentiation of insulin release without stimulatory glucose [38], we found that intracellular argininosuccinic acid concentration was negatively associated with insulin secretion. The discrepancy of these results may result from the concentration and duration of Arg administration. Harmful effects of prolonged L-Arginine exposure at high concentrations on insulin secretion and GSIS of β-cells and islets have been described [22,39], resulting in the concomitant occurring of the increased intracellular concentration of argininosuccinic acid, which is a product of the citrulline-argininosuccinate-arginine cycle, and decreased insulin secretion. This phenomenon also partly explains the negative correlation between L-Citrulline concentration and HGSIS.

Similar to our results that linoleic acid in INS-1 β-cells was correlated with HGSIS, Lai et al. demonstrated that linoleic acid infusion in rat pancreas significantly increased insulin output and GSIS [40]. However, elevated levels of lipid and non-esterified fatty acids were also related to β-cell failure [41]. In our current study, ISCR was positively related to the intracellular 1,2-dioleoyl-sn-glycero-3-phosphatidylcholine concentration, which may be due to the protective effects of phosphatidylcholine in lipotoxicity-induced β-cell dysfunction [42]. Overall, our measurements and analyses provided an important clue for future research regarding effective therapeutic targets for diabetes mellitus.

## 5. Conclusions

Compared with Arg and Lys, excessive His treatment had more significant effects on mitochondrial key enzyme expressions and metabolite profiling in β-cells, which was accompanied by higher insulin secretions. Higher BAA concentrations reduced HGSIS. These results demonstrated metabolism-secretion coupling under BAA administrations and the adverse effects of high concentration exposure. Multiple regression analyses identified potential key genes and metabolites associated with insulin responses induced by BAA treatments. More knowledge of the regulation on β-cell insulin release and the function of these molecules is required to clarify metabolism-secretion coupling and finally explore attractive biomarkers or effective therapeutic targets for diabetes mellitus.

## Figures and Tables

**Table 1:** Primer sequences used in the qPCR ^1^.

Gene	Accession No.	Primers Position	Primers Sequence (5′→3′)
*Abcc8*	NM_013039.2	Forward	CAAGGTCGTAAACCGCAAGC
		Reverse	AGGGGTCCAGGTGAAGAAGC
*Acaca*	NM_022193	Forward	CGGCTGTGGAAATTGCG
		Reverse	GAGGCGGATGGGAATCG
*Actb*	NM_031144.3	Forward	TGTCACCAACTGGGACGATA
		Reverse	GGGGTGTTGAAGGTCTCAAA
*Cs*	NM_130755	Forward	GCCCTCAACAGTGAAAGCA
		Reverse	TGGCAATCAGGTCCATACAG
*Gapdh*	NC_005103.4	Forward	GACATGCCGCCTGGAGAAAG
		Reverse	AGCCCAGGATGCCCTTTAGT
*mt-Atp6*	BC059121	Forward	GCGTCTGGAGGACCTGTTGA
		Reverse	CCTCAGGACTGGGGTTTGT
*mt-Nd4l*	EU104720	Forward	TCCCAATTACCATTCTAGTTTT
		Reverse	AGGTTTTGTACGTAGTCTGTTCCGT
*Ogdh*	NM_001017461.1	Forward	CCGTGCCCGCTGACATTAT
		Reverse	TCTCCCGAAGAGGAAGTGC
*Pdx1*	NM_022852.3	Forward	CCGCGTTCATCTCCCTTTC
		Reverse	TGCCCACTGGCTTTTCCA
*Slc2a2*	NM_181090.2	Forward	GCTGATCTTCATCCTTCCGTCTGC
		Reverse	CCAATCATCACGACTACGCCACTC
*Slc38a2*	NM_181090.2	Forward	GCTGATCTTCATCCTTCCGTCTGC
		Reverse	CCAATCATCACGACTACGCCACTC
*Slc7a7*	NM_031341.1	Forward	TACCTACGCTGGAAGGAACCTGAC
		Reverse	GCGATGCCGATGCCGATGAG
*Tfam*	NM_031326.1	Forward	GAAGAGCAAATGGCTGAAGTT
		Reverse	GTGCCCAATCCCAATGAC
*Ucp2*	NM_019354.2	Forward	TGTGGTAAAGGTCCGCTTCC
		Reverse	TTCGGGCAACATTGGGAG

^1^ *Abcc8*, ATP binding cassette subfamily C member 8; *Acaca*, acetyl-CoA carboxylase alpha; *Actb*, actin, beta; *Cs*, citrate synthase; *Gapdh*, glyceraldehyde-3-phosphate dehydrogenase; *mt-Atp6*, ATP synthase 6, mitochondrial; *mt-Nd4l*, NADH dehdrogenase 4L, mitochondrial; *Ogdh*, oxoglutarate dehydrogenase; *Pdx1*, pancreatic and duodenal homeobox 1; *Slc2a2*, solute carrier family 2 member 2; *Slc7a7*, solute carrier family 7 member 7; *Slc38a2*, solute carrier family 38 member 2; *Tfam*, transcription factor A, mitochondrial; *Ucp2*, uncoupling protein 2.

**Table 2:** Regression analyses for relations between gene expressions and insulin processing in INS-1 β-cells stimulated by different basic amino acids ^1^.

Dependent Variable	Independent Variable	RC	*p*	R^2^
Insulin secretion, ng/10^6^ cells	*Slc7a7*	791.10	0.0023	0.67
	*Pdx1*	−313.65	<0.0001	
	*Tfam*	−1030.39	0.011	
Insulin content, nmol/mg protein	*Cs*	−947.78	0.0065	0.32
	*Nd4l*	639.79	0.038	
ISCR	*Cs*	0.057	0.014	0.24
LGSIS, ng/10^6^ cells	*Slc7a7*	−82.13	0.011	0.26
HGSIS, ng/10^6^ cells	*Slc38a2*	−155.19	0.0009	0.53
	*Ogdh*	−67.19	0.011	

^1^ ISCR, insulin secretion to content ratio; LGSIS, low glucose-stimulated insulin secretion; HGSIS, high glucose-stimulated insulin secretion; RC, regression coefficient.

**Table 3:** Regression analyses for relations between differentially expressed metabolites and insulin processing in INS-1 β-cells treated with different basic amino acids ^1^.

Dependent Variable	Independent Variable (Peak Value)	RC	*p*	R^2^
Insulin secretion, ng/10^6^ cells	Argininosuccinic acid	−0.00077	0.0074	0.29
ISCR	1,2-dioleoyl-sn-glycero-3-phosphatidylcholine	0.000043	0.0099	0.28
LGSIS, ng/10^6^ cells	Oleic acid	−0.000010	0.042	0.17
HGSIS, ng/10^6^ cells	L-Citrulline	−0.00010	0.0001	0.89
	1-Methylnicotinamide	0.00053	0.0014	
	1,2-Benzenedicarboxylic acid	−0.00079	0.0059	
	Alpha-D-Glucose	0.00021	0.0044	
	Linoleic acid	0.000071	0.017	
	Dehydroabietic acid	−0.0013	0.0019	

^1^ ISCR, insulin secretion to content ratio; LGSIS, low glucose-stimulated insulin secretion; HGSIS, high glucose-stimulated insulin secretion; RC, regression coefficient.

## Data Availability

Metabonomic data are made available in the Supplementary Materials Table S1. The other datasets generated during the current study are available from the corresponding author on reasonable request.

## Supplementary Materials

The following supporting information can be downloaded at: https://www.mdpi.com/article/10.3390/nu15184026/s1, Table S1: The relative expressions of DEMs among the 3 groups.
